# Editorial: Rising Stars: Africa

**DOI:** 10.3389/fchem.2022.851125

**Published:** 2022-01-27

**Authors:** Emmanuel O. Balogun, Tebello Nyokong, Aziz Amine, Shivani Mishra, Eno Ebenso

**Affiliations:** ^1^ Department of Biochemistry, Ahmadu Bello University, Zaria, Nigeria; ^2^ Africa Centre of Excellence for Neglected Tropical Diseases and Forensic Biotechnology (ACENTDFB), Ahmadu Bello University, Zaria, Nigeria; ^3^ Department of Chemistry, Rhodes University, Grahamstown, South Africa; ^4^ Faculty of Sciences and Techniques, Hassan II University of Casablanca, Casablanca, Morocco; ^5^ Nanotechnology and Water Sustainability Research Unit, College of Science, Engineering and Technology, University of South Africa, Johannesburg, South Africa

**Keywords:** medicinal chemistry, green chemistry, synthesis, nanoscience—nanotechnology, electrocatalysis, inorganic chemistry, infectious diseases

**FIGURE 1:**
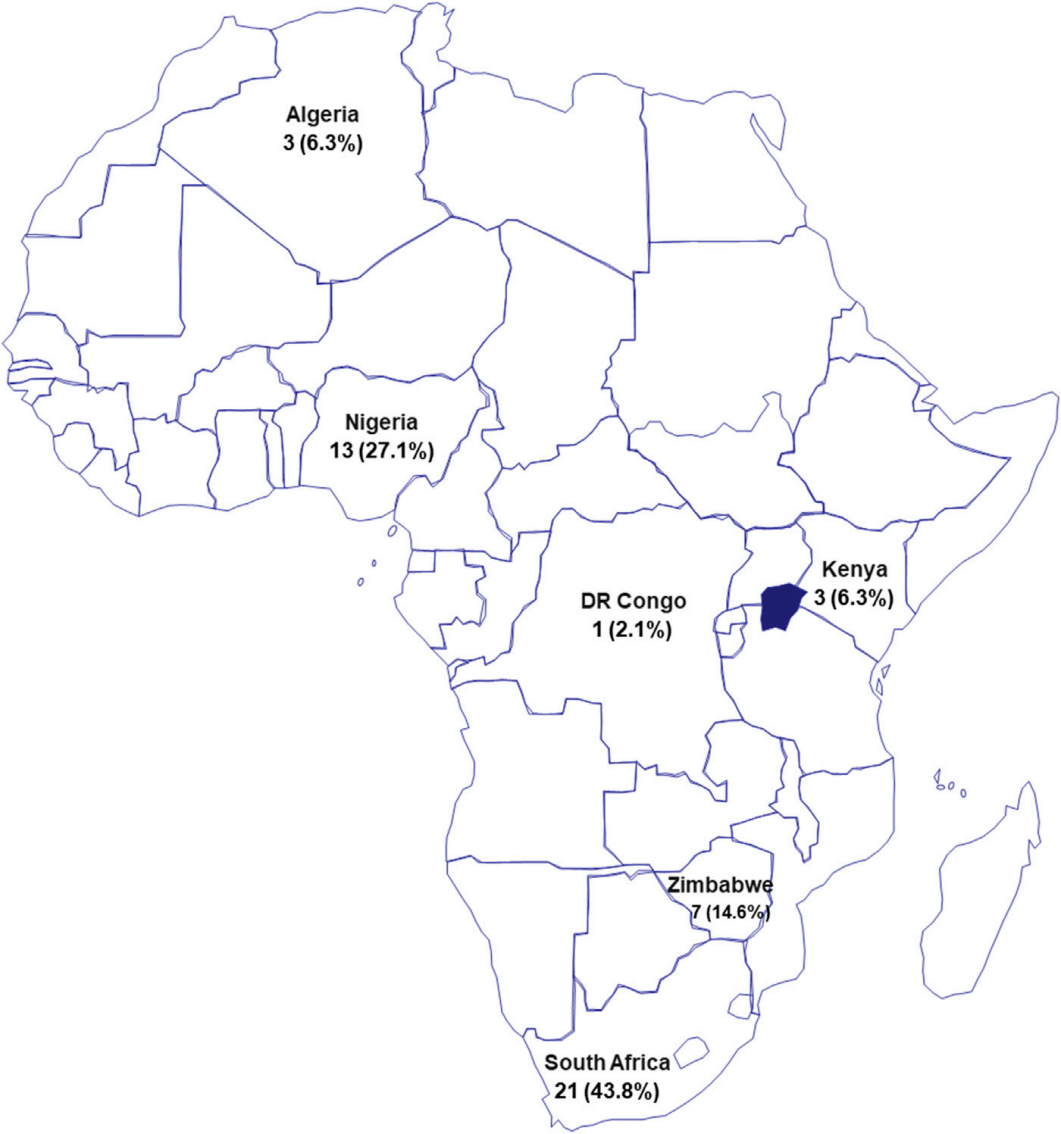
Map of Africa showing the distribution of authors by country of affiliated institution. Authors are from six countries shown in the map. Values represent number of authors and the proportion as percentages in parentheses.

With a total population of about 1.34 billion, Africa accounts for 17.2% of the world population (https://www.worldometers.info/world-population/#region). However, this population does not match research output as Africa contributes less than 1% to global research efforts in terms of scholarly publications from various disciplines of science and engineering as well as the social sciences (Duermeijer et al., 2018). No African country spends up to 0.9% of the gross domestic product (GDP) on research and development (R&D), whereas the four top countries (United States, China, Japan, and Germany) in terms of volume of R&D investment, each commits over 3% of their GDPs to R&D amounting to 613, 525, 173, and 132 billion US$, respectively. Commendably, South Africa is the only African country with annual spending of over $6 billion on R&D, followed by Morocco with $1.5 billion; all other countries in Africa commits less than $1 billion to R&D (OECD, 2022). This annual spending on R&D correlates well with research output. For instance, while 422,808 articles were published by researchers in United States in year 2018; about 13,000 publications emanated from South Africa, and less than 1,000 papers from most African countries. The top performing African countries are Algeria, Egypt, Kenya, Morocco, Nigeria, South Africa, and Tunisia. Noteworthy, there is presently a remarkable improvement of Africa’s contribution to global research output. A recent in-depth analysis using Elsevier’s SciVal tool revealed a consistent positive upturn in Africa’s research productivity. In fact, Africa has been adjudged as having the highest research productivity growth rate of approximately 39% in the past 5 years and this is simultaneously matched with increases in number of authors at an average growth rate of 43% (Duermeijer et al., 2018). Indeed, there are numerous talented African early career scientists who have demonstrated abilities to emerge as regional and global research leaders in their fields. Many of them have received awards, fellowships, and grants that have provided the opportunity to build independent research career and networks across institutions in Africa and shows the potentials to undertake cutting-edge scientific research that will address challenges that are peculiar to the African continent as well as contributing to solve global challenges. These recent advances are highly commendable and encouraging as continuing on this path will help Africa to build a thriving knowledge-based economy and place her as a critical hub for the new industrial revolution. To achieve this goal, the numerous challenges against productive science must be tackled. These challenges include: 1) lack of research facilities, 2) inadequate manpower and skills, 3) lack of national and regional funding mechanisms, 4) poor access to channels for dissemination of research to the global community, and 5) very weak intranational and intracontinental collaborations. To address these limitations, stakeholders comprised of institutions, national governments, and publishers must deploy resources within their means to enhance the successes recorded by African scientists and researchers. This synergy will propel the continent to solve the numerous problems that suppress its economic growth and development. Towards contributing to scientific research in Africa, Frontiers, a leading publisher through one of its journals, *Frontiers in Chemistry* initiated a research topic called “Rising Stars: Africa,” which is a collection to “showcase the compelling work of recognized researchers in the early stages of their independent careers, from the breadth of the African continent.” We believe that this effort will help to tackle challenges #4 and #5 (listed above). The research topic was edited by top African scientists who were carefully selected based on their achievements and contributions to diverse fields of chemistry, which reflects the variety and excellence of the published articles. The works in this collection showcase efforts made by Africans across the chemical sciences and with potential applications to solve compelling problems faced by Africa particularly with regards to infectious diseases, poverty, environmental pollution, energy, and lack of potable water. In this topic, we provide a compilation of the recent progress made by African-led research groups in diverse fields of chemistry, towards addressing African problems. In total there are 14 accepted contributions comprised of 11 original research articles and three review papers all in covering multiple fields: analytical chemistry, biochemistry, chemical synthesis, electrochemistry, green chemistry, immunochemistry, inorganic chemistry, medicinal chemistry, nanotechnology, and physical chemistry. Based on country of affiliations, the contributing African authors are from six countries (listed in order of number of authors); South Africa, Nigeria, Zimbabwe, Algeria, Kenya, and Democratic Republic of Congo (Figure 1).

The articles address numerous regional and global problems that fall under one of these three broad categories- Climate change, environmental pollution, and infectious diseases. The number of papers in these categories were four, three, and seven, respectively, which may reflect the present magnitude of the impacts of these problems on Africa.

Climate change (CC) is a product of ecosystem imbalance and is described as a significant variation in the usual pattern of Earth’s average weather conditions, global warming, changing precipitation patterns, and extreme weather and climate events such as flood and drought, are prominent manifestations (Balogun et al., 2016; WMO 2021). Since emission of carbon dioxide CO_2_ is a major cause CC, strategies to reduce CO_2_ release and/or atmospheric accumulation will help to mitigate the impacts of CC. In this compilation, Tshuma et al described their effort to develop metal-organic frameworks (MOFs), one of the potent techniques for CO_2_ capture and storage (CCS). The successfully developed two MOFs- [Cd2 (bpydc)2 (DMF) 2·2DMF]n (JMS-3) and [Zn (bpydc) (DMF)·DMF]n (JMS-4) from 2,2’-bipyridine-4,4’-dicarboxylate (bpdc) linker and tested their CO_2_ storage and H_2_ adsorption capacity as potential CCS material. Their results revealed that JMS-3a (active form) has better CO_2_ and **H**_2_ adsorption capacity (1.36 and 2.88 mmol·g-1average) than JMS-4a (active form) (0.98 and 2.29 mmol·g-1). However, JMS-3a is not stable under different chemical environments that are normally employed in the catalytic hydrogenation of CO_2_. Thus, making JMS-4a a better potential candidate for application in CCS. The work of Ocansey et al. reported here is highly innovative towards reducing atmospheric CO_2_. There effort was not only in the line of utilizing MOFs for CCS, they have established a catalytic system for enhanced conversion of CO_2_ to useful industrial product, formic acid *via* iridium (III) tetrazole Complex-mediated hydrogenation of CO_2_. The iridium (III) tetrazole Complex catalyst was highly efficient with just 1 μmol producing up to 180 mM concentrated formate solution. Formic acid has wide range applications due to its antibacterial pesticidal property. The worth of annual global consumption of formic acid stands at $680 million. Furthermore, since the use of fossil fuels for energy generation is a chief source of CO_2_ release, tapping and conversion of solar energy has gained wide acceptance as an eco-friendly means of reducing fossil fuel burning. This is made possible by the invention of solar cells, which trap photons from the Sun and convert them to electricity. To create better efficiency solar cells, scientists exploited the intense light absorption and chemical stability of metallophthalocyanines (molybdenum disulfide, MoS_2_ and copper phthalocyanine, CuPc) to produce solar cells (Manamela et al.). The work by Manamela et al. was the first to demonstrate that combination of MoS_2_ and CuPc can efficiently function as a photovoltaic (PV) system. The currently used PV technologies are silicon-based solar cells (SBSCs), which makes this technology too costly for wide penetration most especially for most African countries. To curb this problem, dyes have been used as sensitizers in the fabrication of highly efficient dye-sensitized solar cells (DSSCs) and even the best photosensitizer dye, tricarboxy–terpyridyl ruthenium complex is still far less efficient than the conventional SBSCs. With consideration for low cost and sustainability, organic PVs have been considered and developed from natural sources. In this collection, Sanusi et al., successfully extracted an apigenin derivative from Hibiscus rosa-sinensis plant and validated it as a model for testing the performance of organic PVs as light harvesters in solar cells.

Environmental pollution is the release of harmful or potentially harmful substances (contaminants), which may be solid, liquid or gas into the environment. It is caused by man’s activities through exploration, industrialization, mining, and urbanization. Environmental pollution is the greatest problem facing humanity because it annually causes over nine million human deaths and about $4.6 trillion loss to global economy. Recently, water remediation technology using multi-walled carbon nanotubes (MWCNTs) was developed as an adsorbent of contaminants from water bodies. To improve the efficiency of MWCNTs, Makhado and Hato synthesised sodium alginate/poly (acrylic acid)/oxidized-multi-walled carbon nanotubes hydrogel nanocomposite (SA/p (AAc)/o-MWCNTs HNC) and tested its efficacy on methylene blue contaminated water. They found that SA/p (AAc)/o-MWCNTs HNC had high adsorptive capacity of 1,596.0 mg/g, and importantly, it can be repeatedly used for several cycles without significant reduction in the adsorption capacity. In addition to the use of adsorptive materials for cleaning contaminated water bodies, chemical methods involving photocatalysis (PC) and photoelectrocatalysis (PEC) are in use as safe and efficient techniques. While these techniques are effective for colourless contaminants, their efficiency is compromised when the contaminant is a dye because dyes prevent penetration of light, which is a necessity for PC and PEC actions. To alleviate this problem, Nwachukwu and Arotiba conducted a review of possible solutions to this problem and suggested the use of perovskite oxide–based PC and PEC for highly effective remediation of contaminated water because perovskite possess exceptional optical and electrochemical properties, which makes them capable of efficient light absorption even in the visible region. In addition, perovskite have long-term stability. For effective remediation of contaminated soil and water it is important to have a robust quantitative analytical method. In the context of Africa, the method should be simple, cheap, and highly efficient. The present analytical tools such as chromatography and mass spectrometry do not fulfil the stated criteria. Therefore, Zouaoui et al. developed a molecularly imprinted chitosan film grafted on a 4-Aminophenylacetic Acid on gold electrode as a microsensor for accurate detection and quantification of glyphosate, which is a generally used herbicide and a common contaminant of soil and water. Their microsensor is very sensitive. It can detect the contaminant at a concentration as low as 1 × 10^–12^ mg/ml (Zouaoui et al.).

Africa, particularly the sub-Saharan countries bear the highest impacts of infectious diseases. The five topmost killer infectious diseases in Africa are acute respiratory infections, HIV/AIDS, diarrhoea, malaria, and tuberculosis. They are responsible for more than six million deaths per year. These diseases are a major reason why life expectancy in sub-Saharan Africa is as low as 46 years compared to 85 years for Japan (Boutayeb, 2010). The economic impact of diseases to Africa is estimated as $2.4 trillion/year (WHO 2019). For Africa to overcome this problem, concerted efforts are needed with respect to intervention development for new, cheap, simple, and effective diagnostics, vaccines, and/or drugs. At present, most diagnostic techniques such as the enzyme-linked Immunosorbent Assays (ELISA) are expensive, time-consuming, expensive, requires bulky equipment, and highly skilled personnel for performing the analysis and result interpretation. Owing to these, the use of electrochemical immunoassay (EI) was developed to overcome the ELISA drawbacks. Using C-reactive protein, Adesina and Mashazi fabricated immunosensor for impedimetric detection by covalently immobilising oriented antibody on gold electrodes. In addition to significantly increased sensitivity, results from the immunosensor can be digitized. Tuberculosis is one of the major infectious diseases in Africa. Although isoniazid and pyrazinamide are first line drugs for treatment, toxic side effects and reduced efficacy are associated drawbacks. Towards solving this problem, Ngilirabanga et al. employed a novel approach to co-crystallize the drugs isoniazid and pyrazinamide with glutaric acid. Their results showed that there was an improvement of desirable physicochemical properties (increased solubility and stability) of the drug co-crystals, which may consequently improve their pharmacological and therapeutic properties. Two papers, a Review by Marzuq et al. and a research article by Anyam et al. provided a comprehensive insight into efforts by African scientists to combat tropical infectious diseases through search for novel natural compounds for development of chemotherapy. Marzuq et al. catalogued medicinal chemistry information on 93 compounds from different natural sources that have been described to possess antiparasitic activities against the pathogens of malaria, trypanosomiasis, and leishmaniasis. Such detail information on the different novel drug candidate compounds in a single piece, will enhance antiparasitic drug development. The discovery of medicinal phytochemicals complements high-tech drug discovery programs involving screening of combinatorial libraries and rational drug discovery (Balogun et al., 2019), which may not be practicable in the continent due to limited technologically advanced facilities. Resistance to drugs by pathogenic organisms is a major problem in disease management by chemotherapy. Drug resistance may result from genetically encoded resistance factors such as drug degrading enzymes or drug efflux transport proteins, or through formed extracellular matrices that enable agglutination to form a community (most especially bacteria) and colonise surfaces. The last scenario reduces the accessibility of an administered drug to the bacteria, therefore, preventing the drug effect. With this background, Openda et al. developed a photodynamic antimicrobial chemotherapy technique (PACT) in which metal-conjugated porphyrins are loaded onto carbon nanomaterials and used to treat *Staphylococcus aureus* followed by exposure to light. They reported that the treatment was effective on both planktonic and biofilm forms. The mechanism of action of PACT is based on nanomaterial-mediated rapid transport of conjugated photosensitiser dyes across barriers and metal-enhanced photosensitiser property of the dyes (such as porphyrins), i.e., ability to generate injurious reactive species upon exposure to light. Recent advances in PACT were adequately reviewed by Oyim et al. where they concluded on the possibility of adding PACT systems to the current therapeutic arsenals for combating microbial resistance, especially where the conventional antimicrobials have failed. Finally, it is important continue investigations toward development of more sensitive analytical techniques for detection and quantitation of pollutants, contaminants, or drugs in both the environment and in living systems. In this regard, based on the specific electrocatalytic behaviour of chemical substances, Chundu et al. successfully developed and fabricated an electrochemical sensor for accurate detection and quantification of various analytes. This was achieved through co-polymerization of different monomeric forms of phthalocyanine and conjugated to graphene oxide, and finally linked to glassy carbon electrode. They validated the sensor by using it detect and quantify ascorbic acid and tryptophan, two coexisting physiologically important molecules.

In conclusion, this research topic has provided an excellent platform for African scientists to share their research and achievements with the global research community. We believe this will promote collaborations amongst scientists in the continent towards bringing solutions to the myriads of problems that affect the continent and the world at large.

The guest editors acknowledge the authors for their commendable efforts, as well as the reviewers for their time and dedication. We sincerely thank the editorial staff of this issue for this bold idea and valuable assistance throughout the editing process.
